# Microencapsulation of Olive Oils from Nizip and Kilis Yağlik Varieties by Freeze-Drying: Effects of Wall Materials on Physicochemical Properties and Bioactive Compounds

**DOI:** 10.3390/foods15061044

**Published:** 2026-03-16

**Authors:** Eda Elgin Kiliç, Songül Kesen

**Affiliations:** Naci Topcuoglu Vocational School, Gaziantep University, 27600 Gaziantep, Turkey

**Keywords:** olive oil, freeze-drying, microencapsulation, phenolic compounds, antioxidant activity, physical property

## Abstract

This study investigated the characteristics and bioactive properties of olive oils obtained from regional Nizip Yaglik (NY) and Kilis Yaglik (KY) olive varieties, encapsulated using maltodextrin (MD) and whey protein isolate (WPI) as wall materials. Olive oils were first emulsified with different WPI–MD ratios (1:1, 1:4, 1:10) and subsequently freeze-dried to produce microcapsule powders. A comprehensive evaluation was conducted, including physicochemical properties (encapsulation efficiency, moisture content, water activity, bulk density, flowability, wettability, particle size, and color), FTIR spectral profiles, morphological features, total phenolic content, and antioxidant activity. The results demonstrated that combining WPI with MD yielded high encapsulation efficiency and favorable reconstitution characteristics, effectively protecting sensitive bioactive constituents from oxidative degradation during processing and storage. Increasing the proportion of MD in the wall matrix improved emulsion stability and microencapsulation yield, while also slightly enhancing powder brightness. FTIR analyses confirmed that the fundamental chemical structure of olive oil was preserved across all formulations. The freeze-dried microcapsules displayed superior stability relative to non-encapsulated oils, retaining higher levels of phenolic compounds and antioxidant capacity. Among the formulations, elevated MD ratios enhanced powder flowability, whereas WPI played a crucial role in emulsification performance and capsule surface integrity. Overall, these findings underscore the effectiveness of MD–WPI blends as promising wall materials for the freeze-drying encapsulation of regional olive oils, offering a viable strategy to preserve their distinctive qualities and bioactive potential for functional food applications.

## 1. Introduction

Olive oil is derived from the fruit of the olive tree (*Olea europaea* L.). It is extensively cultivated in Spain, Italy, Tunisia, Greece, and Turkey. Olive oil contains antioxidant, anti-inflammatory, and antibacterial effects [1]. Olive oil is known for its rich phenolic content and the preservation of these compounds is critical for the retention of its health benefits [2]. Nizip olive oil and Kilis olive oil, the main olive varieties in Southeastern Anatolia, are grown in the Gaziantep, Kahramanmaraş, and Mardin provinces of Türkiye. Their fruits are small, their pits are large, and they contain 27–31% oil. They are generally used in olive oil production. Their oils have a balanced and fruity aroma. However, very little research has been conducted on the phenolic composition and antioxidant properties of Nizip olive oil and Kilis olive oils. In the literature, it is reported that the total phenolic content of the oils varies depending on the varieties, place of origin, agricultural techniques, and olive ripening [3]. A study showed that the phenolic compound content of NY and KY oils varied depending on both the variety and the year [4].

Microencapsulation is a technological process in which a core material (e.g., lipids or bioactive compounds) is enclosed within one or more coating matrices to preserve the functional integrity of sensitive food components. This technique enhances protection against oxidative degradation, improves physicochemical stability, and allows for controlled release of the encapsulated substances [5]

Microencapsulation of olive oil is a widely used technique to preserve the quality of the oil, prevent oxidative deterioration, and increase its usability in various applications. Microencapsulation significantly increases the oxidative stability of olive oil, which is essential for preserving its health benefits and sensory properties [6]. Additionally, encapsulated olive oil can be incorporated into functional foods aimed at providing health benefits such as improved antioxidant properties and anti-inflammatory effects [7].

Freeze-drying, also known as lyophilization, is a method used for microencapsulation, particularly for heat-sensitive and volatile substances. A key advantage of freeze-drying is its operation at low temperatures. This reduces the thermal degradation of sensitive bioactive chemicals, preserving their structural integrity and functionality throughout the drying process [8].

In microencapsulation processes, the emulsion plays a critical role as an intermediate system where the active compound (e.g., essential oil, phenolic compound, aroma, etc.) is combined with the wall material to form the capsule core. The droplet size, homogeneity, and stability of the emulsion formed directly affect the microcapsule morphology, encapsulation yield, and release properties of the final product. In the microencapsulation, the selection of wall material is one of the most critical steps affecting microencapsulation efficiency, physicochemical properties, and the storage stability of the microencapsulated powder. The choice of wall material combinations affects both the emulsion properties and the properties of the particles after drying and during storage. It is well known that emulsion properties such as stability, viscosity, droplet size, and powder properties such as surface oil, particle size, density, morphology, and oxidative stability are affected by the type of encapsulation agent used [9].

Maltodextrin is widely used as a carrier agent in the food industry and is also an important wall material in microencapsulation processes. Its low viscosity in solution, ease of dissolving in water, neutral taste and odor, and capacity to create a good coating that protects encapsulated oil droplets from oxidation are the primary factors contributing to its preference [10]. However, it shows poor emulsion stability and emulsifying abilities. Therefore, it is desirable to use maltodextrin in combination with other materials to provide functional and effective microencapsulation. The combined use of modified whey proteins and maltodextrin was reported to improve capsule surface properties and increase microcapsule yield up to 89% [11]. Previous studies have investigated the microencapsulation of virgin olive oils [12], virgin coconut oil [13], olive paste [14], and essential oils [15] with different wall materials. However, there are only a limited number of studies on the production of freeze-dried microcapsules of olive oil using WPI and MD [16,17]. Calvo et al. [16] used carboxymethylcellulose and maltodextrin as wall materials, while Chaabane et al. [17] obtained freeze-dried powdered oil microcapsules using different ratios of wall material (WPI:MD) compared to those used in this study.

During the microencapsulation process, the phenolic content of oils varies significantly depending on the type of oil used. The phenolic compound content of each oil and the protection and stability properties these compounds provide during microencapsulation may differ. For example, phenols in some oil types are classified as more polar compounds and may be more effective in providing long-term stability during microencapsulation, while others exhibit less polar properties, making protection more difficult [18,19]. The use of different wall material ratios in the microencapsulation process affects the bioavailability of microencapsulated products [20]. Adjusting the wall material ratios in the microencapsulation process of oils has a significant effect on the protection, stability, and bioavailability of phenolic compounds. The wall material ratios used in microencapsulation are also important in terms of optimizing microencapsulation strategies.

Thus, the aim of this study was to obtain powder microcapsules for the first time by freeze-drying oils obtained from Nizip Yağlık and Kilis Yağlık olive varieties. In the microencapsulation process, WPI and MD, used as wall materials, were used for the first time in combination ratios of 1:1, 1:4, and 1:10 (WPI:MD), and the effects of these ratios on the encapsulation efficiency (ME), physicochemical properties (particle size, fluidity, color, morphology), phenolic compounds, and antioxidant activity (AA) of microcapsules were determined.

## 2. Materials and Methods

### 2.1. Material

The Nizip Yaglik (NY) and Kilis Yaglik (KY) olive varieties used in the study were harvested in their own region (Nizip and Kilis) in December 2024 and oil production was carried out in plants using two-phase continuous oil extraction technology (Oliomio mini, Florence, Italy). Whey protein isolate (WPI, Protein Ocean, Ankara, Turkey), maltodextrin (MD, Alfasol, Kocaeli, Turkey) used as wall material and Tween 80 (Merck, Darmstadt, Germany) used as a stabilizer were obtained from suppliers. All chemical reagents used were of analytical grade.

### 2.2. Method

#### 2.2.1. Preparation of Emulsion

Microencapsulation of olive oil is achieved by the core material (oil) forming a fine and stable emulsion in the wall solution. The emulsion was formed from different proportions of maltodextrin (MD) and whey protein isolate (WPI) as wall material. Emulsions were prepared with different proportions (weight basis) of WPI and MD. In all formulations, the oil content in the emulsion was set at 12.5 g and the water content at 62.5 g (Table 1). The emulsion was prepared by homogenization using a mechanical homogenizer (Ultra Turrax T18, IKA, Staufen im Breisgau, Germany) at 167 mHz for 300 s. A mixture of oil and Tween 80 was added dropwise to the wall material during homogenization at 333 mHz for 600 s to ensure successful homogenization [12]. The ratio of oil to wall material in the emulsion was 1:2.

#### 2.2.2. Determination of Emulsion Stability

The stability of the emulsion was assessed using the methodology outlined by Carneiro et al. [21]. The produced emulsions were transferred to 25 mL measuring cups, sealed with plastic wrap, and maintained at room temperature for 24 h. The height of the upper phase of the emulsion was measured after 24 h. The stability of the emulsion was determined as “% separation” using the following equation.

%Separation = (H_1_/H_0_) × 100

H_0_: The initial emulsion height; H_1_: The upper phase’s height after 24 h

#### 2.2.3. Drying Process

The emulsion was directly frozen in Falcon tubes at −80 °C for 24 h before being connected to the freeze-drying unit. Drying was conducted for 48 h utilizing a freeze dryer (Labconco, Kansas City, MO, USA) at a vacuum of 0.1 mbar and a condenser temperature of −52 °C. Microparticles generated through freeze-drying were stored in a refrigerator at 4 °C.

#### 2.2.4. Determination of Microencapsulation Efficiency

The efficiency of microencapsulation is evaluated by determining the ratio of oil, excluding surface oil, to the total oil contained in the powder product obtained from the microencapsulation process. To determine the quantity of surface oil, the powdered sample was introduced into a beaker, combined with petroleum ether, and then filtered. The resulting residue was reconstituted with petroleum ether, subjected to further filtration, and subsequently heated in an oven at 70 °C to evaporate the petroleum ether until a stable weight was achieved. The Petri dish was measured, and the quantity of surface oil was assessed. The total volume of oil in the equation is presumed to be equivalent to the oil added in relation to the original volume of the carrier [22].

ME (%) = (TOA − SOA)/TOA

ME: Microencapsulation Efficiency; TOA: Total Oil Amount (g); SOA: Surface Oil Amount (g).

### 2.3. Physicochemical Analysis of Microcapsules

#### 2.3.1. Moisture Content and Water Activity

The moisture contents of the microcapsules were evaluated using an infrared moisture analyzer (XM 120; Precisa Instruments Ltd., Dietikon, Switzerland) and the water activity (aw) values were determined using a water activity meter (Rotronic Hygropalm, Bassersdorf, Switzerland) at 25 °C.

#### 2.3.2. Particle Size of Microcapsules

Particle size analysis of microcapsules produced under optimum conditions was performed using the HORIBA Laser Scattering Particle Size Distribution Analyzer LA-950 (Horiba Ltd., Kyoto, Japan). Particle size analysis of the encapsulated oil sample was measured in a pure water matrix (1:50 ratio). During the particle size measurements, the refractive index and density values were set according to the default parameters of the instrument software. The refractive index of olive oil at 20 °C was assumed to be 1.4453, density 0.91775 g/cm^3^ and refractive index of pure water 1.3330. These values were taken from the Horiba LA-950 instrument database. The average diameter was determined based on the average diameter of a sphere of the same volume, which is generally used to characterize powder particles. Particle size was expressed as De Brouckere mean diameter D_(4.3)_ [23].

De Brouckere Mean = D_(4.3)_ = Ʃ NiDi4/Ʃ NiDi3

The Di value is the geometric mean of the diameters (square root of the upper*lower diameters) and Ni is the number of particles with Di diameter in the emulsion.

‘Span’ values, which give the homogeneity of the distribution, were calculated with the equation given below. D_(10)_, D_(50)_, and D_(90)_ were diameters at 10, 50, and 90 percent cumulative volume, respectively [24].

Span = [D_(90)_ − D_(10)_]/D_(50)_

#### 2.3.3. Bulk and Tapped Density

The bulk density of about 2 g (m_0_) of microencapsulated olive oil powder was calculated from the mass/volume ratio by filling a cylindrical container of 10 mL volume without air pockets and without applying any pressure [25]. The tapped bulk density was calculated from the mass-to-volume ratio of the powder in the cylinder after 100 taps on a bench [26].

Bulk Density (ρB) (g/mL) = m_0_/V_0_

Tapped density (ρT) (g/mL) = m_0_/V_n_

V_0_: Volume in the cylinder

V_n_: Sample volume in the same cylindrical container at tapped density

#### 2.3.4. Flowability

The flowability and stickiness values of powdered products are determined according to the Carr Index (CI) and Hausner Ratio (HR). The Carr index (CI) was calculated using bulk density and tapped density values (CI = ((ρT − ρB)/ρT) × 100) [27]. The bulk density and tapped density of powder products categorize the flowability as follows: CI value below 15 indicates very good flowability, 15–20 indicates good flowability, 20–35 indicates poor flowability, 35–45 indicates poor flowability and a value exceeding 45 indicates very poor flowability.

The Hausner Ratio (HR) was calculated using bulk density and tapped density values (HR = ρT/ρB) [28]. HR values categorize the stickiness as follows: HR value below 1.2, the stickiness is considered low; between 1.2 and 1.4, considered medium; and above 1.4, considered high.

#### 2.3.5. Wettability

For the assessment of the wettability of the powder sample, 100 mL of distilled water placed in a 250 mL beaker, maintaining a temperature of 25 °C (±1 °C). The total wetting time for 1 g of the powder sample was recorded [28].

#### 2.3.6. Particle Morphology

Scanning electron microscopy was used to evaluate the morphological structures (particle structures and pores) of the microencapsules. High vacuum, 5 kV and 200× magnification were used to generate SEM images using a Scanning Electron Microscope (Carl Zeiss, Oberkochen, Germany) after palladium coating of the microencapsulated samples to improve image clarity.

#### 2.3.7. Color Properties

L* (brightness value), a*± (redness index) and b*± (yellowness index) values of mi-croencapsulated powders produced by freeze-drying in a lyophilizer were evaluated during storage (on days 0, 45, 90) using HunterLab ColorFlex EZ (HunterLab, Reston, VA, USA) [29].

#### 2.3.8. Fourier-Transform Infrared Spectroscopy (FTIR)

Fourier-transform infrared spectroscopy (FTIR) scan of samples was carried out on an FTIR spectrophotometer (IRAffinity-1S, Shidmadzu Corporation, Kyota, Japan), operating between 4000 and 400 cm^−1^ with a resolution of 1 cm^−1^ at room temperature. The sample was read directly with Attenuated Total Reflectance (ATR).

### 2.4. Determination of Phenolic Compounds

Phenolic compound extraction was carried out using the International Olive Oil Council’s recommended methodology [30]. One milliliter of syringic acid, the internal standard, was added to a test tube containing two grams of oil sample. A total of 5 mL of methanol + water (80/20, *v*/*v*) was added after 30 s of mixing, and the mixture was vortexed for 1 min. After 15 min in an ultrasonic bath, it was centrifuged for 25 min at 5000 rpm. After collecting the upper phase, the extracts were filtered through 0.45 μm membrane filters and then added to the HPLC. ChemStation software (version B.04.03, Agilent Technologies, Santa Clara, CA, USA) was utilized to operate an Agilent 1100 high performance liquid chromatography (HPLC) system (Agilent Technologies, Palo Alto, CA, USA). The diode array detector (DAD) was utilized in conjunction with the HPLC apparatus. A Beckman C18 ODS (Roissy, France) column measuring 4.6 mm by 250 mm by 5 μm was utilized, along with a precolumn measuring 4.6 mm by 10 mm by 5 μm. Our earlier research clarified these analysis techniques [4]. Using an Agilent 6430 Triple Quadrupole LC/MS System spectrometer (Agilent Technologies, Palo Alto, CA, USA), each compound was identified and assigned by comparing its retention times and UV spectra to genuine standards. This was further verified by mass spectrometry (liquid chromatography coupled with electrospray ionization tandem mass spectrometry). An external standard calibration curve for each detected constituent was used to quantify the compounds on oil and powder samples. The phenolic compounds in microencapsulated powders produced by freeze-drying in a lyophilizer were evaluated during storage (on days 0, 45, and 90).

### 2.5. Determination of Antioxidant Activity

To determine antioxidant activity DPPH method was performed. 3.9 mL of DPPH solution (2.36 mg/100 mL methanol) was combined with 0.1 mL of diluted olive oil extract, and the mixture was violently vortexed. For fifteen minutes, the solution was kept at room temperature in the dark. An ultraviolet visible spectrophotometer (Shimadzu UV-1201, Kyoto, Japan) was used to detect the absorbance at 517 nm. The antioxidant activity of the extracts was determined using the Trolox calibration curve, and the antioxidant capacity was expressed in µmol Trolox equivalent per gram of sample [4]. The antioxidant activity values of microencapsulated powders produced by freeze-drying in a lyophilizer were monitored during storage (on days 0, 45, and 90).

### 2.6. Statistical Analysis

The findings obtained as a result of the analyses were subjected to analysis of variance using SPSS 22 package program and significant differences were evaluated according to Duncan multiple comparison test. The differences and/or similarities in the data collected during storage were categorized according to the initial source and evaluated using Principal Component Analysis (PCA), a multivariate data analysis method [31].

## 3. Results and Discussion

### 3.1. Emulsion Stability of Microcapsules

The percentage of separation of emulsions produced with different proportions of wall material, which expresses the emulsion stability, is given in Table 2. Optimizing consistent emulsion stability is essential for emulsion efficiency. The stability of an emulsion pertains to the physical stability of emulsion systems, enabling the emulsion to maintain homogeneity over extended periods. A high computed percentage of emulsion separation signifies instability, whereas a low percentage denotes great stability.

The stability of an emulsion is indicated by its computed percentage of separation; a low percentage of separation suggests great stability, whereas a large percentage of separation implies instability.

At the end of 24 h at room temperature, separation (%) of the emulsions varied between %10.45–11.55. (Table 1). These results indicate that a low separation rate (%) indicates high emulsion stability. The effect of wall material ratio on emulsion stability after 24 h is statistically significant (*p* < 0.05).

In the emulsion formulations obtained as a result of microencapsulation of both varieties (NY; KY) of olive oil with MD and WPI wall materials, it was observed that the separation percentage decreased with an increase in the MD ratio (from 1:1 to 1:10). This result shows that the stability of the emulsion increases with an increase in the MD ratio. Similarly, Matsumura et al. [32] explained the positive effect of maltodextrin, which has little or no surface active properties, on emulsion stability by the viscous nature of the maltodextrin solution, which prevents the aggregation of fat globules in the emulsion. At the end of 24 h, it is seen that the separation percentage results obtained are consistent with the separation percentages obtained from the results of studies with different core materials and wall materials [33,34]. The study determined that the stability of emulsions varied depending on the ratios of the components in the formulation.

### 3.2. Microencapsulation Efficiency (ME) of Microcapsules

To evaluate the influence of wall materials on the microencapsulation efficiency, the amounts of surface oil and total oil of the powders were determined. The surface oil is crucial for the oxidative stability of the powder as the surface oil may oxidize more easily in the presence of oxygen [13]. The microencapsulation efficiency of olive oil powders obtained from different varieties of olive oil microencapsulated with MD and WPI is presented in Table 3. The ME value, which varies according to the wall composition, ranges from 87.09 to 90.56 and 87.30 to 90.59 for NY and KY olive oil powders, respectively. In previous studies, microencapsulation efficiency values by freeze-drying were reported for fish oil (29.40–81.60%) [35]; flaxseed oil (32.68–59.63%) [36]; olive oil (36.90–69.09%) [16], and rosemary essential oil (69.90–96.14%) [37]. The significant differences in microencapsulation efficiency values noted by different researchers, despite the usage of freeze-drying for all oils, can be attributed to the different types and ratios of wall materials employed, as well as the core-to-wall material ratio.

According to the analysis results of microencapsulation efficiency, an increase in the MD ratio in the wall material had a positive effect on microencapsulation efficiency. The highest microencapsulation value in olive oil powder obtained from the microencapsulation of both varieties of olive oil was determined in olive oil powders (NY, KY) at a ratio of 1:10 (WPI:MD). The effect of the MD ratio in the wall material on the microencapsulation efficiency mentioned in the study has also been similarly stated in the studies by Calvo et al. [16] and Koç et al. [12]. The microencapsulation efficiency values of powdered oils obtained by freeze-drying microencapsulated oils were found to be statistically significant (*p* < 0.05).

### 3.3. Physicochemical Properties of Olive Oil Microcapsules

#### 3.3.1. Moisture Content and Water Activity Values of Microcapsules

Moisture content and water activity values are important factors during the storage of powdered products [38], because high moisture content leads to oil oxidation and affects the flowability of the powdered product. Moisture content and water activity are also important for preventing microbial activity during storage. In the food industry, the maximum acceptable moisture content for many dry products is between 3 and 4% [39]. To ensure microbial stability in foods, the water activity value should be below 0.6 [40,41]. Moisture content also affects the processability properties of powdered products, such as flowability and wettability [42]. The changes in moisture content of microencapsulated olive oils (NY, KY) containing different ratios of wall material on days 0, 45, and 90 are presented in Table 4. The moisture content of olive oil microcapsules obtained by freeze-drying is between 2.33% and 2.86% at the start of storage. The moisture content of microencapsulated powders produced from NY and KY olive varieties, respectively, varied between 2.33% and 3.21% and between 2.26% and 3.29% for different formulation ratios during storage (Table 4). It has been determined that moisture content increases during storage and depending on the MD ratio of the wall material. Chaabane et al. [17] noted that, as in our study, the change in lyophilized olive oil powders was similar with different wall material ratios.

Water activity values for microencapsulated NY and KY olive oil with different formulation ratios were determined to be between 0.02 and 0.31 and 0.01–0.35, respectively, at 25 °C. The water activity of the microcapsules on the 90th day was higher than the 0th day storage period in all formulations. The change in water activity value during storage was statistically significant for microcapsules (*p* < 0.05). During storage of microencapsulated oils containing different proportions of wall materials, water activity values are below this critical value. In the literature, the water activity of microcapsules obtained by freeze-drying is reported to be 0.22–0.27 for fish oil [43], 0.036 for annatto seed oil [34] and 0.04–0.06 for sunflower oil [35].

#### 3.3.2. Particle Sizes of Microencapsules

The particle size and density of food powder are critical considerations because these factors affect appearance, flowability, and reconstitution. They are important for evaluating the overall stability and delivery properties of the microencapsulated product. The D_(4,3)_ value, also known as the volume-weighted mean diameter, represents the average particle size in the distribution of microencapsulated particles. The mean particle size D_(4,3)_, D_10_, D_90_, and median D_50_ particle sizes and dispersion values (span) of the different samples are given in Table 5.

As seen in Table 5, the average particle sizes ranged from 11.02 to 12.54 µm. For both microencapsulated oil types, microcapsules with a 1:10 (WPI:MD) ratio had the largest particle size. The span values of microencapsulated powders of olive oil ranged from 0.20 to 0.27. The increase in span value indicates an increase in the space between particles. Although all wall material combinations resulted in similar particle size distributions, the powders produced with the WPI:MD (1:10) mixture showed a wider distribution, indicating that these particles were less homogeneous [44]. It is thought that the increase in particle size in the formulations has a positive effect on antioxidant properties by providing a greater barrier against oxygen diffusion to the active ingredient encapsulated within the microcapsule. As particle size increases, the exposed surface area per unit volume decreases, thereby limiting oxygen diffusion and contact with the encapsulated oil. This physical effect contributes to the enhanced oxidative stability observed in larger particles, in addition to the barrier properties of the polymeric wall materials [45].

**Figure 1:**
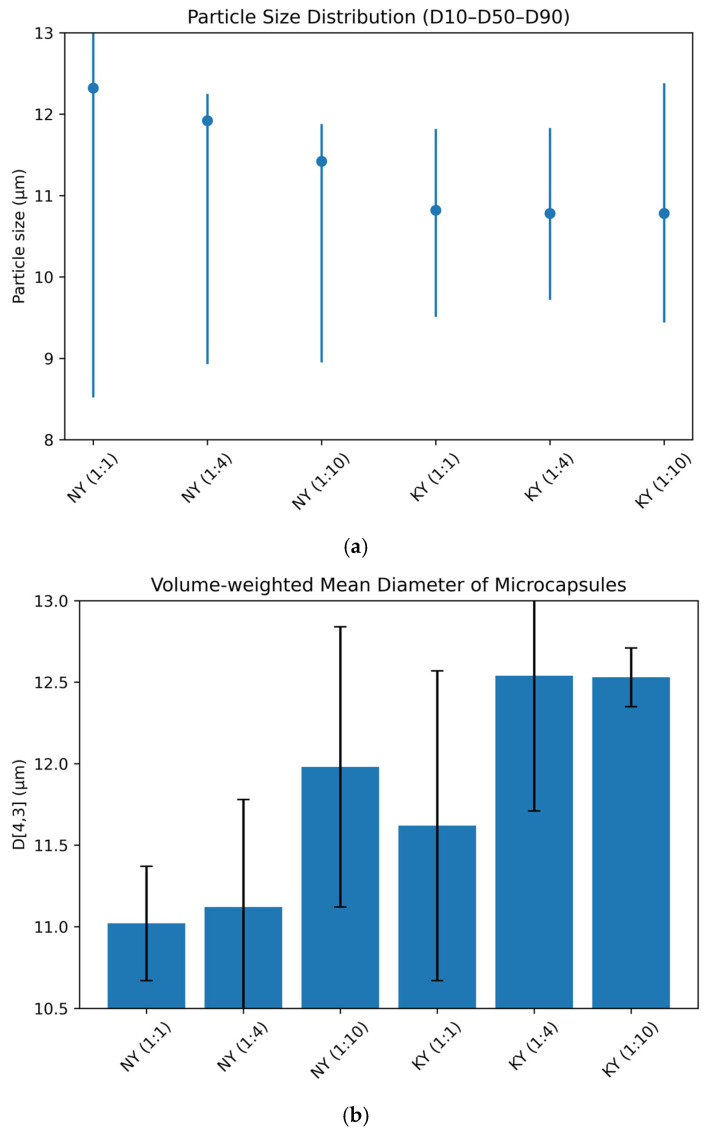
Particle size distribution parameters (D_10_–D_50_–D_90_) (**a**) and volume-weighted mean diameter (D_(4,3)_) (**b**) of NY and KY microcapsules prepared at different wall material ratios.

Graphical visualization of particle size parameters is presented in Figure 1a,b. As shown in Figure 1a, all NY and KY microcapsules exhibited relatively narrow particle size distributions, with D_50_ values in the micrometric range. Differences in distribution width among formulations are clearly observed through the D_10_–D_90_ intervals, which is consistent with the Span values reported in Table 5. Figure 1b shows the volume-weighted mean diameter D_(4,3)_, providing a volume-based comparison of particle size and supporting the numerical results.

#### 3.3.3. Bulk and Tapped Density of Microcapsules

The bulk density (ρB), tapped density (ρT) and flowability of microencapsulated oil powders were determined and the results were presented in Table 6. During storage, bulk properties are important physical properties for the characterization of food powders. Low bulk density is not desirable as it increases packaging volume. Furthermore, products with low bulk density contain more air in the spaces between them, which increases the risk of oxidation; this also reduces the storage stability of the product [21]. The bulk density values showed a very slight increase for each microencapsulated olive oil (0.25 to 0.26 g/cm^3^) at the WPI:MD (1:10) wall material ratio, and there was no statistically significant change between the bulk densities of the samples (*p* > 0.05). The advantage of obtaining higher-density powders is that they can be stored in larger quantities in smaller containers compared to lower-density products [46]. In studies using maltodextrin and protein-based coating materials using different drying methods [21,36,47], the bulk density values were found to be close to the bulk density values obtained in the present study**.** Similarly, Bae and Lee [23] reported that the bulk densities of spray-dried avocado oil microcapsules ranged from 0.25 g to 0.28 g/cm^3^ and that the bulk density increased with increasing maltodextrin content in the formulation.

The tapped density value was measured to calculate the Carr Index and Hausner ratio data, which provide an idea about the flowability of the powder products. A large increase in the tapped density value due to compression is an indicator of possible flowability problems that may be observed in the powder product [48]. In the present study, the increase in the density values of the powders due to compression varied between 52 and 60. For these values, the flowability properties of the obtained microcapsules are expected to be poor. The tapped density values of the microcapsules obtained in the study were observed to be between 0.38 and 0.40 g/cm^3^ (Table 6). The effect of the wall material composition on the tapped density values of the microcapsules is not significant (*p* > 0.05).

#### 3.3.4. Flowability of Microcapsules

The flowability properties of a product can be determined using various empirical methods such as Carr’s Index (CI), rebound angle, Hausner ratio (HR) or compression test [49]. In this study, the flowability properties of powders were investigated using Carr’s Index and Hausner ratio values. The classification of the flowability and stickiness of powdered products according to CI and HR has been determined based on the study by Jinapong et al. [28]. The Carr’s Index and Hausner ratio values of microcapsules containing different olive oil varieties showed that the effects of different wall material ratios were statistically insignificant (Table 6). Carr’s Index values ranged between 35.24 and 36.60; Hausner ratio values ranged between 1.54 and 1.58 for oil samples. It was observed that CI and HR values increased with increasing MD ratio in the microcapsule formulation of both types of oil samples. This can be confirmed by the presence of low molecular weight saccharides in the MD, as microcapsules with higher MD ratios are more hygroscopic and cohesive [50,51]. The flow properties of the microcapsules obtained were found to be poor, and previous studies have also indicated that the flow properties of encapsulated oils are poor [44,52].

#### 3.3.5. Wettability of Microcapsules

The ability to rehydrate in water or the capacity of a bulk powder to absorb water is known as wettability [53]. This facilitates the penetration and passage of water into the powder. In this study, wettability was determined by measuring the time it takes for particles to disappear from the water surface (Table 6).

The study found that the wetting times of the microcapsules obtained ranged from 61 to 80 s. It was observed that an increase in the amount of MD in the wall material shortened the wettability time.

This indicates that MD, which is more hydrophilic than WPI, increases the hydrophilicity of the wall systems. The wettability values of wall materials NY (1:1) and KY (1:1) were found to be higher and statistically different (*p* < 0.05) than those of other wall materials. It has also been noted in the study by Bae and Lee [23] that the maltodextrin content strengthens the hydrophilic structure of the microcapsules, facilitating water penetration between the particles and thus shortening the wetting time.

#### 3.3.6. Morphology of Microcapsules

**Figure 2:**
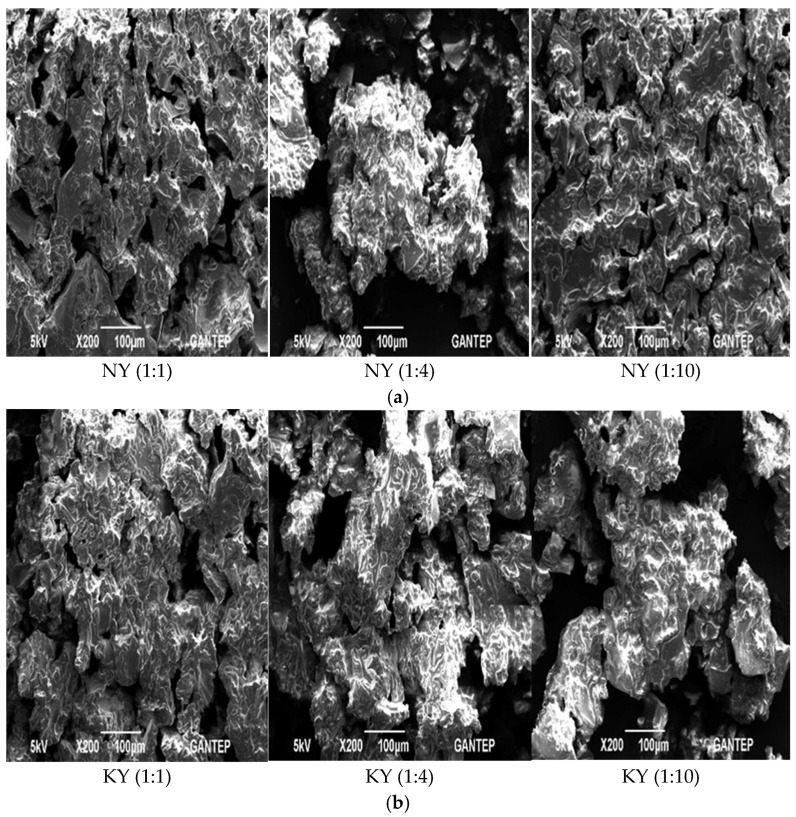
Scanning electron microscope (SEM) images × 200 of microencapsulated olive oil powders of NY (**a**) and KY (**b**) with different wall material ratios (WPI:MD).

Scanning electron microscope images of the ground microcapsules after freeze-drying are presented in Figure 2. It was determined that the microcapsules containing olive oil had an irregular shape and a rough, porous, perforated, and occasionally fractured surface structure (Figure 2).

The irregular shape and rough pore structure of the olive oil microcapsules obtained in this study are typical characteristics of products dried in a lyophilizer [54]. During freeze-drying, a decrease in ambient pressure causes the water in the emulsion to crystallize and the frozen water to sublimate at the minimum temperature [55]. This affects the structure and integrity of the wall materials of the microcapsules obtained by freeze-drying and causes a porous structure to form [56]. This porous structure is thought to originate from the voids left behind by ice crystals that form during freezing and then vaporize through sublimation [57]. The morphology images obtained in this study show a similar porous, irregular structure to the freeze-dried fish oil microcapsule [43], olive oil-lemon juice microcapsule [58], and tea tree essential oil [59].

#### 3.3.7. Color Values of Microcapsules During Storage

The average Hunter L, a*, and b* values of the samples during storage are given in Table 7. The L* values of each product are high. This indicates that the products are white and bright. The L* values in NY oil show a change of up to 0.75% (from 89.46 to 89.36) from day 0 to day 90. On the other hand, the L* value of KY oil increased by 1.01% (from 87.16 to 89.15).

It was observed that the increase in maltodextrin slightly increased the brightness of the microcapsules, and the change in the L* value was found to be statistically significant (*p* < 0.05). The a* values of all powders were negative, indicating a tendency towards the green color range. On day 0, the values of the NY powders ranged from −0.02 to −0.40, while the values of the KY powders ranged from −0.22 to −0.49, indicating a slightly more intense green tone in the KY samples. During storage, a slight change from red to green was observed in all samples. The greatest change was observed in the KY (1:10) formulation, with a change from −0.49 to −0.94. A statistically significant difference was observed in the a* values between days 0 and 90. Statistically significant changes were observed in the b* values, which indicate the color cycle between yellow (+) and blue (−), during storage (*p* < 0.05). A decrease in b values was observed depending on the storage period (between 0 and 90 days).

#### 3.3.8. FTIR Spectroscopy Analysis of Microcapsules

**Figure 3:**
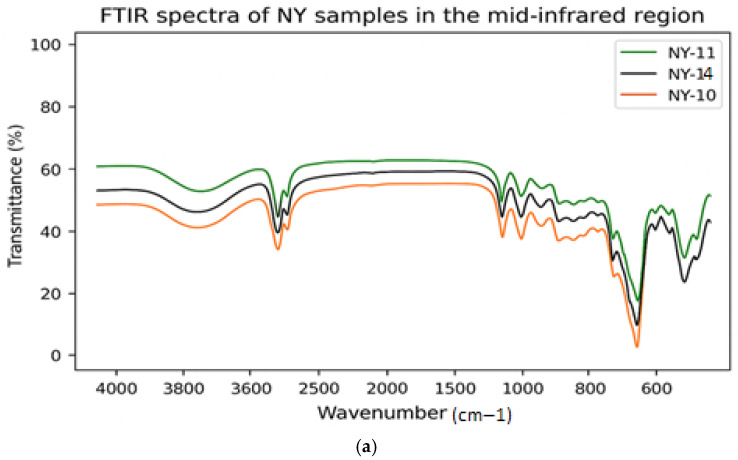

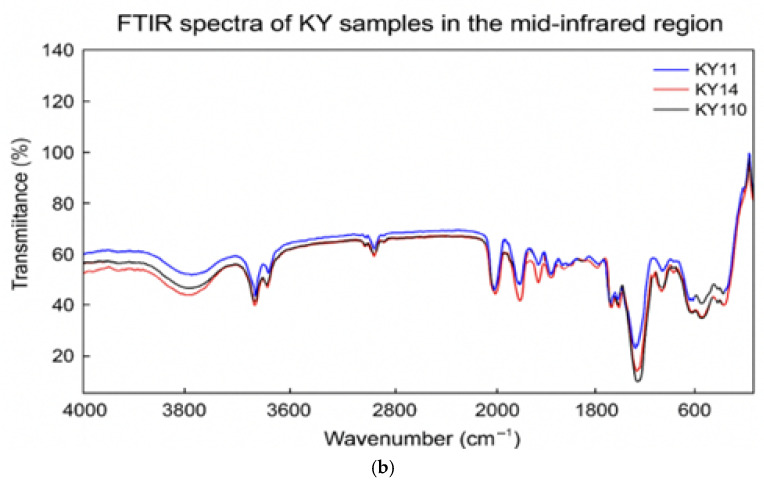
Fourier transform infrared (FTIR) analysis of freeze-dried NY (**a**) and KY (**b**) samples (NY (1:1): green; NY (1:4): orange, NY (1:10): black; KY (1:1): blue; KY (1:4): red; KY (1:10): black).

Fourier transform IR (FTIR) analysis (400–4000 cm^−1^) was performed to identify functional groups and assess structural stability of microencapsulated olive oils (NY and KY). As shown in Figure 3 (a: NY, b: KY), both spectra retained characteristic olive oil bands—such as =C–H (3006 cm^−1^), –CH_2_/–CH_3_ (2922–2853 cm^−1^), C=O (1743 cm^−1^), aliphatic bending (1465–1377 cm^−1^), and long-chain rocking (722 cm^−1^) confirming preservation of the lipid structure after encapsulation and freeze-drying. Differences between NY and KY were evident: NY samples showed stronger O–H (3600–3200 cm^−1^), Amide I–II (~1650, ~1540 cm^−1^), and carbohydrate (1200–900 cm^−1^) bands, indicating higher protein and maltodextrin contributions. Within both groups, 1:1 ratios showed pronounced protein bands, while 1:10 ratios exhibited stronger carbohydrate signals. Overall, the FTIR results revealed that although the core olive oil structure remained intact, NY formulations demonstrated more effective protein–carbohydrate interactions during encapsulation.

#### 3.3.9. Phenolic Component of Microcapsules

**Figure 4:**
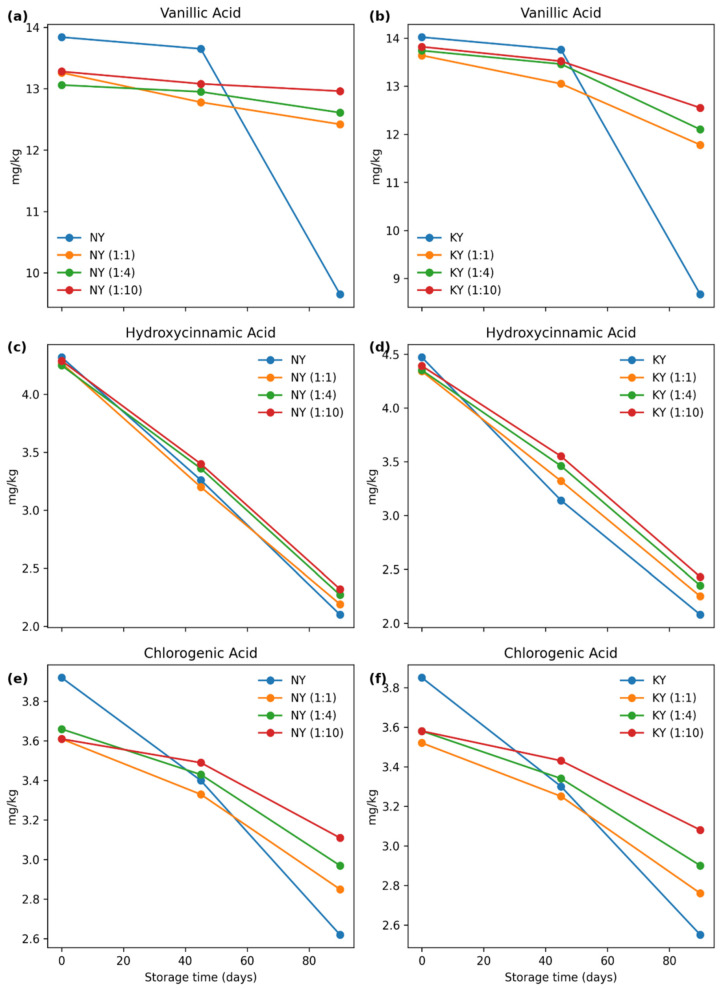
Changes in selected phenolic compounds during storage in NY and KY samples. Vanillic acid, hydroxycinnamic and chlorogenic acid contents (mg/kg) are shown for NY (**a**,**c**,**e**) and KY (**b**,**d**,**f**) samples and their microencapsulated formulations (1:1, 1:4, and 1:10) measured at 0, 45, and 90 days of storage. The multi-panel representation highlights the time-dependent degradation of key phenolic compounds and the protective effect of increasing wall material ratios in both sample types.

Table 8 and Table 9 show the amounts of phenolic compounds determined after encapsulation of NY and KY olive oils in various formulations (1:1, 1:4, 1:10), and the changes that occurred during storage (0, 45, and 90 days). It is noted that differences in the stability of phenolic compounds during encapsulation vary depending on the encapsulation structure and pretreatment methods [60]. To facilitate the visual interpretation of these changes, representative phenolic compounds (vanillic, hydroxycinnamic and chlorogenic acids) that have the highest concentrations in microcapsule samples, show significant changes with storage time and clearly reflect the preservative effect of microencapsulation ratios (1:1, 1:4, 1:10) are shown in Figure 4. Vanillic acid, which was determined to be the highest among phenolic compounds in NY and KY olive oils at 13.84 mg/kg and 14.02 mg/kg, respectively, showed higher values on day 0 in all formulations, although it decreased during storage. However, by day 90, a significant decrease was observed, particularly in non-encapsulated oils, with values of 9.65 mg/kg for NY and 8.67 mg/kg for KY. This trend, also clearly visualized in Figure 4, can be attributed to the inherent instability of phenolic compounds under long-term storage conditions, where oxidative degradation plays a major role in phenolic loss [61]. Studies show that phenolic compounds such as vanillic acid are particularly susceptible to oxidation during storage [62]. Among the formulations, the best protection of vanillic acid during storage (at the end of 90 days) was observed in NY (1:10) and KY (1:10) with values of 12.96 mg/kg and 12.55 mg/kg, respectively. A similar trend is observed for hydroxycinnamic acid in Figure 4; a regular decrease over time is seen in all samples, while the decrease is slower in encapsulated samples. In the case of chlorogenic acid, the highest loss was again determined in the control samples. The most stable profile was observed in the 1:10 formulation for both oils. A similar situation was observed in both oil samples for the quercetin and luteolin compounds. A previous study indicated that flavonoids such as quercetin and luteolin have higher stability rates throughout storage periods [63]. It was observed that all phenolic compounds decreased during storage, but the decrease was less compared to non-encapsulated oils. The formulation providing the best protection for all compounds in both oil samples was determined to be 1:10. This situation is thought to be due to an increase in maltodextrin concentration. Previous studies have also indicated that maltodextrin is important in the microencapsulation of phenolic compounds in formulations, increasing their stability and protection [63,64,65,66,67]. Mecha et al. [68] stated that phenolic interactions with wall materials used in formulations during encapsulation processes can modulate antioxidant properties, and that the synergistic effects of various wall materials can increase the retention of compounds during storage.

#### 3.3.10. Antioxidant Activities of Olive Oil and Microcapsules

**Figure 5:**
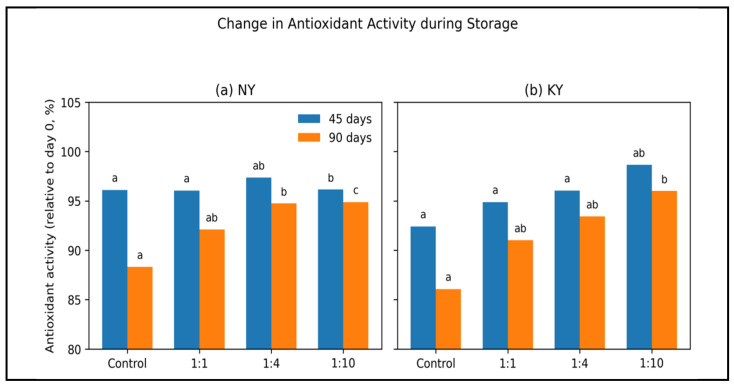
Percentage change in antioxidant activity of NY (**a**) and KY (**b**) olive oils during storage. Antioxidant activity values are expressed as percentage relative to day 0 (100%) for non-encapsulated oils and microencapsulated formulations (1:1, 1:4, and 1:10) measured after 45 and 90 days of storage. The bar charts highlight the effect of storage time and encapsulation ratio on antioxidant activity retention. Different lowercase letters above the bars indicate significant differences among treatments (*p* < 0.05). Bars sharing the same letter are not significantly different.

The antioxidant activity (mM TE/kg) values in microcapsules obtained using olive oil and different wall material ratios are shown in Table 10 throughout the storage periods (0, 45, and 90 days). To compare storage-induced changes among the samples, antioxidant activity was expressed as a percentage change and visualized in Figure 5. A significant decrease in antioxidant activity values was observed throughout the storage period in NY and KY oil samples and in all microcapsule formulations. This decrease was statistically significant for each sample during storage (*p* < 0.05). As clearly illustrated in Figure 5, the decrease in antioxidant activity during storage is related to the oxidation of phenolic compounds and potential degradation processes [69,70].

The decrease in antioxidant activity of NY and KY oils during storage was 11.51% and 13.62%, respectively, while the decrease in antioxidant activity in microencapsulated oils was 8.18%, 5.85%, and 5.61% for NY (1:1), NY (1:4), and NY (1:10) formulations, respectively, and 8.30%, 5.87%, and 4.87% for KY (1:1), KY (1:4), and KY (1:10) formulations. The reduction in antioxidant activity following the freeze-drying process was similarly noted by Franceschinis et al. [71] in the microencapsulation of blackberry juices with maltodextrin, as well as by Hee et al. [13] in virgin coconut oil microparticles composed of a blend of maltodextrin, gum arabic, sodium caseinate, and whey protein concentrate. However, it has been determined that microcapsules with the NY (1:10) and KY (1:10) formulations exhibit higher antioxidant activity during storage as shown in both Table 10 and Figure 5. Therefore, it has been determined that the 1:10 (WPI:MD) wall material has the potential to provide better protection. The addition of MD to the wall system has been observed to have a positive effect on oxidative stability. This may be partly due to oxygen being hydrophobic and the presence of the more hydrophilic MD potentially reducing the oxygen permeability of the wall matrix [72]. A study demonstrating the positive effect of using carbohydrates such as maltodextrin in wall materials on oxidative stability indicated that a wall matrix composed solely of protein has much higher oxygen permeability than a wall matrix composed of a mixture of protein and low molecular weight carbohydrates [23].

#### 3.3.11. PCA Results of the Phenolic Component of Olive Oil and Microcapsules

**Figure 6:**
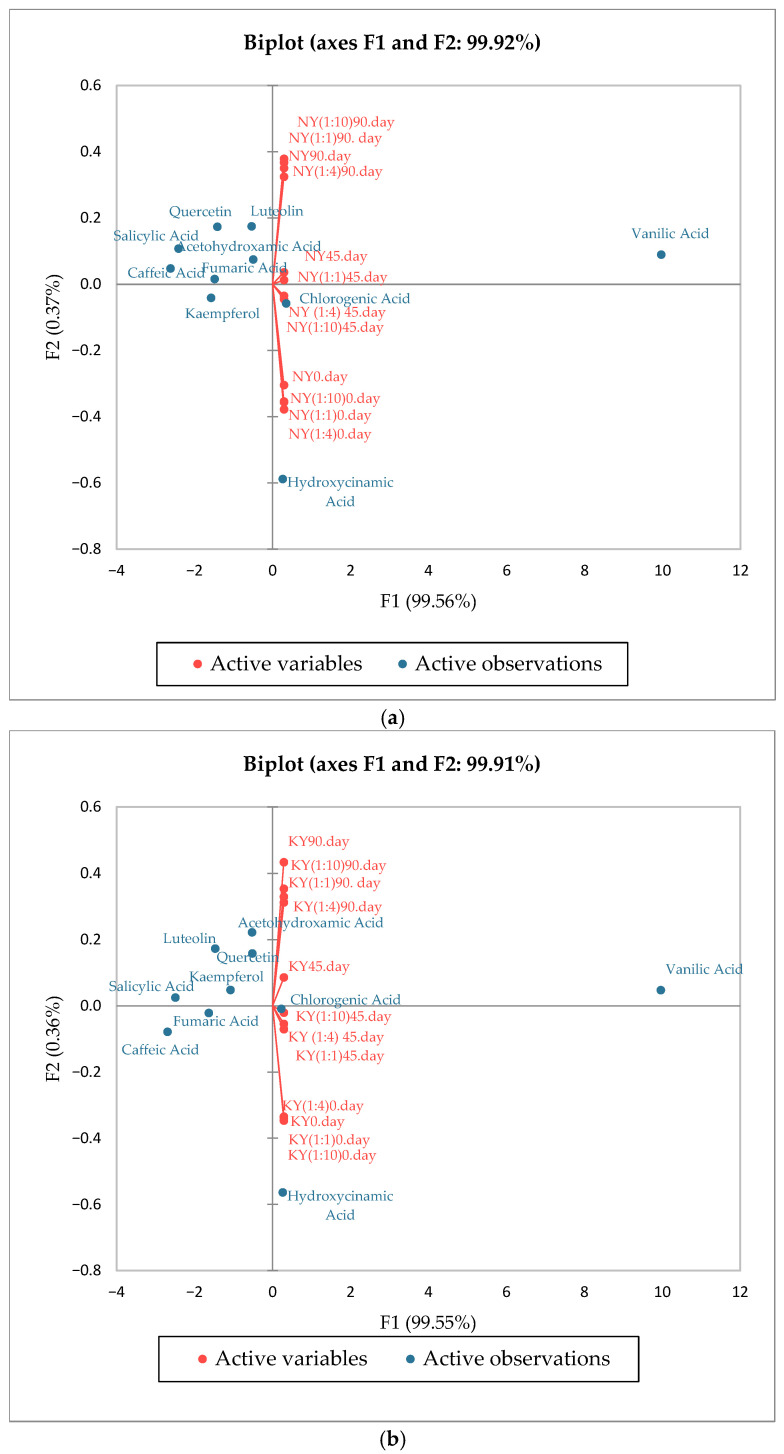
Principal Component Analysis (PCA) biplot based on phenolic profiles of NY (**a**) and KY (**b**) at different WPI:MD ratio and storage time.

Principal Component Analysis was performed to investigate the relationships among phenolic compounds and the changes in their profiles across different treatments and storage times (Figure 6). PCA of NY and KY sample groups revealed similar overall trends. For the NY samples, the first two principal components (PC) explained 99.92% of the total variance, with PC1 explaining 99.56% and PC2 explaining only 0.37%. For KY samples, as seen in the graph, explains 99.91% of the total variance, with PC1 explaining 99.55% and PC2 explaining only 0.36%. This indicates that most of the variance in the dataset is captured by the first axis. In both biplots, a clear temporal distinction was observed between the 0-, 45-, and 90-day samples, indicating significant changes in phenolic composition during storage. In both groups, day 0 samples clustered on the negative side of PC1, while day 90 samples were shifted towards the positive side, indicating marked changes in phenolic composition over time. Vanillic acid was consistently associated with longer storage periods and showed a strong positive correlation with PC1 in both datasets. Conversely, hydroxysuccinic acid exhibited a strong negative loading on PC1 in both cases.

## 4. Conclusions

The findings clearly indicate a synergistic relationship between the maltodextrin (MD) ratio and the stability and efficiency of the microencapsulation system. Increasing the MD ratio from 1:1 to 1:10 resulted in lower separation percentages, thereby enhancing emulsion stability. For both olive oil varieties, the highest microencapsulation efficiency was achieved at a 1:10 (WPI:MD) ratio. Over the course of storage, the moisture content of the microencapsulated powders exhibited an increasing trend, which corresponded with a significant rise in water activity by day 90. These changes highlight the sensitivity of the microcapsules to moisture accumulation over time. Particle size analysis revealed that the 1:10 formulation produced the largest particle sizes. Bulk density values showed minimal variation across different wall material ratios and were not statistically significant (*p* > 0.05). However, Carr Index and Hausner ratio values suggest that the flowability of the microcapsules remains relatively poor. Wettability assessments demonstrated reduced wetting times with increasing MD concentrations. Color analysis indicated high L* values for both oil samples, suggesting good brightness retention. Importantly, storage led to a reduction in phenolic compounds and antioxidant activity; however, these decreases were significantly less pronounced in the encapsulated oils compared to the non-encapsulated counterparts. The 1:10 (WPI:MD) formulation offered superior protection of bioactive compounds, preserving both phenolics and antioxidant capacity more effectively during storage in both NY and KY olive oils. Overall, these results suggest that microencapsulation at a 1:10 (WPI:MD) ratio presents a promising approach for maintaining the quality and functional properties of olive oil in food applications and may contribute to advancements in functional food technologies.

## Figures and Tables

**Table 1:** Compositions of emulsions prepared by NY and KY olive oils with the different proportions of MD and WPI.

	1:1	1:4	1:10
WPI (g)	12.50	5.00	2.27
MD (g)	12.50	20.0	22.73
Oil (g)	12.50	12.50	12.50
Water (g)	62.50	62.50	62.50

**Table 2:** Separation values of emulsions produced with various wall materials after 24 h (%).

Emulsion Formulations of Microcapsules	Separation, % *
NY (1:1)	11.55 ± 0.06 ^e^
NY (1:4)	11.44 ± 0.03 ^d^
NY (1:10)	10.98 ± 0.04 ^b^
KY (1:1)	11.44 ± 0.03 ^d^
KY (1:4)	11.09 ± 0.04 ^c^
KY (1:10)	10.45 ± 0.06 ^a^

* Each analysis was performed in triplicate and the results were reported as mean value ± standard deviation. Separation of emulsions (%) with different lowercase letters (a, b, c, d, e) in the same column are statistically significant (*p* < 0.05).

**Table 3:** Microencapsulation efficiency of microcapsules (%) with different ratio wall materials.

Microencapsules	Microencapsulation Efficiency (ME, %) *
NY (1:1)	87.09 ± 1.17 ^a^
NY (1:4)	89.46 ± 1.62 ^ab^
NY (1:10)	90.56 ± 0.16 ^b^
KY (1:1)	87.30 ± 1.99 ^a^
KY (1:4)	89.33 ± 1.29 ^ab^
KY (1:10)	90.59 ± 0.20 ^b^

* Results are the mean value of three replications along with the standard deviation (mean ± std dev.). The microencapsulation efficiencies of microcapsules indicated by different lowercase letters (a,b) within the same column are statistically significant (*p* < 0.05).

**Table 4:** Moisture content and water activity values of oils encapsulated with different proportions of wall material (WPI + MD) during storage.

Moisture content, %	**Days**	**NY (1:1)**	**NY (1:4)**	**NY (1:10)**	**KY (1:1)**	**KY (1:4)**	**KY (1:10)**
	0	2.33 ± 0.05 ^aA^	2.65 ± 0.02 ^aB^	2.88 ± 0.04 ^aC^	2.26 ± 0.02 ^aA^	2.62 ± 0.04 ^aB^	2.86 ± 0.05 ^aC^
	45	2.48 ± 0.03 ^bA^	2.71 ± 0.02 ^aB^	3.14 ± 0.13 ^bC^	2.44 ± 0.04 ^bA^	2.78 ± 0.04 ^bB^	3.23 ± 0.06 ^bC^
	90	2.50 ± 0.06 ^bA^	2.83 ± 0.08 ^bB^	3.21 ± 0.05 ^bC^	2.49 ± 0.04 ^bA^	2.93 ± 0.03 ^cB^	3.29 ± 0.07 ^bC^
Water activity, a_w_	0	0.02 ± 0.00 ^aA^	0.03 ± 0.01 ^aA^	0.13 ± 0.01 ^aB^	0.01 ± 0.00 ^aA^	0.02 ± 0.01 ^aA^	0.12 ± 0.01 ^aB^
	45	0.08 ± 0.00 ^bA^	0.12 ± 0.01 ^bB^	0.28 ± 0.01 ^bC^	0.11 ± 0.01 ^bA^	0.18 ± 0.01 ^bB^	0.24 ± 0.02 ^bC^
	90	0.19 ± 0.00 ^cA^	0.21 ± 0.01 ^cB^	0.31 ± 0.01 ^cC^	0.26 ± 0.01 ^cA^	0.28 ± 0.01 ^cB^	0.35 ± 0.02 ^cC^

Each analysis was repeated three times, and results are reported as mean ± standard deviation. Different small letters in the columns for each sample during storage time indicate that statistically significant differences in moisture content and water activity (*p* < 0.05) on days 0, 45 and 90. Different large letters on the same storage days indicate statistically significant differences (*p* < 0.05) in water activity and moisture change between formulations.

**Table 5:** Particle size (μm) of NY and KY powder encapsulated with different proportions of wall material (WPI + MD).

Microencapsules	D_(4.3)_	D_10_	D_90_	D_50_ (medyan)	Span
NY (1:1)	11.02 ± 0.35 ^a^	8.52 ± 0.20 ^a^	11.42 ± 0.37 ^a^	12.32 ± 1.04 ^a^	0.24 ± 0.02 ^a^
NY (1:4)	11.12 ± 0.66 ^a^	8.93 ± 0.13 ^b^	11.59 ± 0.21 ^a^	11.92 ± 1.17 ^a^	0.22 ± 0.01 ^a^
NY (1:10)	11.98 ± 0.86 ^a^	8.95 ± 0.04 ^b^	11.88 ± 0.12 ^a^	11.42 ± 0.70 ^a^	0.26 ± 0.02 ^a^
KY (1:1)	11.62 ± 0.95 ^a^	9.51 ± 0.21 ^a^	11.82 ± 0.18 ^a^	10.82 ± 0.15 ^a^	0.21 ± 0.02 ^a^
KY (1:4)	12.54 ± 0.83 ^a^	9.72 ± 0.19 ^a^	11.83 ± 0.41 ^a^	10.78 ± 0.03 ^a^	0.20 ± 0.06 ^a^
KY (1:10)	12.53 ± 0.18 ^a^	9.44 ± 0.30 ^a^	12.38 ± 0.38 ^a^	10.78 ± 0.60 ^a^	0.27 ± 0.01 ^b^

Data were presented as mean with standard deviation of three measurements (*n* = 3). ^a,b^ Different letters in the columns for NY and KY samples (determined separately for each oil sample) indicate statistically significant differences. (*p* < 0.05).

**Table 6:** ρB, ρT, CI and HR values of NY and KY oils encapsulated with different ratios of wall material (WPI + MD).

Microcapsules	(ρB) g/cm^3^	(ρT) g/cm^3^	CI (%)	HR	Wettability (s)
NY (1:1)	0.25 ± 0.00 ^a^	0.38 ± 0.01 ^a^	35.24 ± 1.24 ^a^	1.54 ± 0.03 ^a^	80.00 ± 2.83 ^b^
NY (1:4)	0.25 ± 0.00 ^a^	0.39 ± 0.00 ^a^	36.06 ± 0.92 ^a^	1.56 ± 0.02 ^a^	68.00 ± 5.66 ^a^
NY (1:10)	0.26 ± 0.01 ^a^	0.39 ± 0.01 ^a^	36.60 ± 0.89 ^a^	1.58 ± 0.02 ^a^	63.00 ± 1.41 ^a^
KY (1:1)	0.25 ± 0.00 ^a^	0.38 ± 0.01 ^a^	35.33 ± 1.70 ^a^	1.55 ± 0.04 ^a^	78.00 ± 4.24 ^b^
KY (1:4)	0.25 ± 0.01 ^a^	0.40 ± 0.01 ^a^	36.36 ± 1.26 ^a^	1.57 ± 0.03 ^a^	66.50 ± 2.12 ^a^
KY (1:10)	0.26 ± 0.00 ^a^	0.40 ± 0.00 ^a^	36.59 ± 0.00 ^a^	1.58 ± 0.00 ^a^	61.00 ± 1.41 ^a^

Identical letters in the columns represent no statistically significant differences between the microencapsulated powder values of each olive oil (*p* > 0.05). Each analysis was performed in triplicate and the results were reported as mean value ± standard deviation.

**Table 7:** The change in color values of microencapsulated oils with different wall material ratios during the storage time.

Day	Color	NY (1:1)	NY (1:4)	NY (1:10)	KY (1:1)	KY (1:4)	KY (1:10)
0	L*	89.46 ± 0.01 ^aA^	91.11 ± 0.13 ^abC^	90.14 ± 0.02 ^aB^	87.16 ± 0.08 ^aA^	90.18 ± 0.01 ^aC^	89.57 ± 0.10 ^aB^
	a*	−0.02 ± 0.01 ^bC^	−0.38 ± 0.01 ^cB^	−0.40 ± 0.01 ^bA^	−0.22 ± 0.02 ^cA^	−0.42 ± 0.00 ^cB^	−0.49 ± 0.01 ^cA^
	b*	18.23 ± 0.02 ^aB^	16.92 ± 0.10 ^cA^	18.54 ± 0.09 ^bC^	20.87 ± 0.03 ^abC^	19.15 ± 0.02 ^aA^	20.51 ± 0.12 ^bB^
45	L*	90.02 ± 0.42 ^bA^	91.49 ± 0.46 ^bB^	91.68 ± 0.20 ^cB^	89.15 ± 0.06 ^bA^	90.63 ± 0.07 ^bC^	90.47 ± 0.01 ^bB^
	a*	−0.26 ± 0.05 ^aC^	−0.64 ± 0.04 ^bA^	−0.45 ± 0.01 ^aB^	−0.15 ± 0.04 ^bC^	−0.57 ± 0.01 ^bB^	−0.80 ± 0.03 ^bA^
	b*	18.35 ± 0.18 ^bB^	16.57 ± 0.14 ^bA^	16.60 ± 0.16 ^aA^	21.41 ± 0.17 ^bC^	19.84 ± 0.06 ^bA^	20.68 ± 0.44 ^bB^
90	L*	89.36 ± 0.13 ^aA^	90.86 ± 0.27 ^aB^	90.85 ± 0.15 ^bB^	89.15 ± 0.48 ^bA^	91.12 ± 0.19 ^cB^	90.72 ± 0.10 ^cB^
	a*	−0.22 ± 0.03 ^aC^	−0.74 ± 0.08 ^aA^	−0.47 ± 0.01 ^aB^	−0.28 ± 0.03 ^aC^	−0.68 ± 0.01 ^aB^	−0.94 ± 0.02 ^aA^
	b*	17.35 ± 0.08 ^cC^	16.11 ± 0.11 ^aA^	16.67 ± 0.13 ^aB^	20.18 ± 0.13 ^aC^	19.08 ± 0.29 ^aA^	19.50 ± 0.16 ^aB^

Results are the mean values of three replications and standard deviation (mean ± std dev.) of color values. Color values (L*, a*, b*) of microcapsule samples with different lowercase letters (a–c) in the same column are statistically significant (*p* < 0.05) during storage time for each samples. Color values of microcapsule samples with different capital letters (A–C) in the same row are statistically significant for the same day (*p* < 0.05).

**Table 8:** Phenolic compounds of NY olive oil and microcapsules during storage (0, 45, 90 days) (mg/kg).

Phenolic Compounds (mg/kg)	NY			NY (1:1)			NY (1:4)			NY (1:10)		
	0. Day	45. Day	90. Day	0. Day	45. Day	90. Day	0. Day	45. Day	90. Day	0. Day	45. Day	90. Day
Vanilic Acid	13.84 ± 0.11 ^bA^	13.65 ± 0.44 ^bA^	9.65 ± 0.12 ^aA^	13.26 ± 0.21 ^aA^	12.78 ± 0.37 ^aA^	12.42 ± 0.78 ^aB^	13.06 ± 0.45 ^aA^	12.95 ± 0.98 ^aA^	12.61 ± 0.83 ^aB^	13.28 ± 0.36 ^aA^	13.08 ± 0.31 ^aA^	12.96 ± 0.65 ^aB^
Acetohydroxamic Acid	2.86 ± 0.04 ^bB^	2.64 ± 0.11 ^abB^	2.38 ± 0.15 ^aA^	2.72 ± 0.14 ^bAB^	2.51 ± 0.07 ^abAB^	2.26 ± 0.11 ^aA^	2.44 ± 0.14 ^bB^	2.32 ± 0.11 ^bA^	2.02 ± 0.08 ^aA^	2.75 ± 0.11 ^aAB^	2.63 ± 0.13 ^aB^	2.35 ± 0.16 ^aA^
Kaempferol	1.87 ± 0.04 ^cB^	1.16 ± 0.00 ^bA^	1.03 ± 0.03 ^aA^	1.66 ± 0.04 ^bA^	1.32 ± 0.06 ^aAB^	1.17 ± 0.05 ^aAB^	1.74 ± 0.08 ^bAB^	1.45 ± 0.09 ^aBC^	1.24 ± 0.08 ^aBC^	1.78 ± 0.04 ^bAB^	1.56 ± 0.13 ^abC^	1.35 ± 0.04 ^aBC^
Chlorogenic Acid	3.92 ± 0.10 ^cA^	3.40 ± 0.14 ^bA^	2.62 ± 0.10 ^aA^	3.61 ± 0.12 ^bA^	3.33 ± 0.13 ^bA^	2.85 ± 0.19 ^aAB^	3.66 ± 0.28 ^bA^	3.43 ± 0.12 ^abA^	2.97 ± 0.21 ^aAB^	3.61 ± 0.24 ^aA^	3.49 ± 0.24 ^aA^	3.11 ± 0.16 ^aC^
Fumaric Acid	1.74 ± 0.04 ^bA^	1.57 ± 0.16 ^bA^	1.10 ± 0.04 ^aA^	1.69 ± 0.09 ^bA^	1.61 ± 0.04 ^bA^	1.25 ± 0.09 ^aAB^	1.68 ± 0.04 ^bA^	1.63 ± 0.05 ^bA^	1.33 ± 0.06 ^aB^	1.71 ± 0.12 ^bA^	1.69 ± 0.07 ^bA^	1.41 ± 0.05 ^aB^
Caffeic Acid	0.65 ± 0.01 ^cA^	0.21 ± 0.01 ^bA^	0.14 ± 0.01 ^aA^	0.52 ± 0.01 ^cA^	0.46 ± 0.02 ^bB^	0.33 ± 0.02 ^aB^	0.51 ± 0.03 ^bA^	0.48 ± 0.02 ^bB^	0.36 ± 0.01 ^aB^	0.53 ± 0.09 ^aA^	0.48 ± 0.03 ^aB^	0.42 ± 0.03 ^aC^
Hydroxycinamic Acid	4.32 ± 0.06 ^cA^	3.26 ± 0.08 ^bA^	2.10 ± 0.04 ^aA^	4.27 ± 0.13 ^cA^	3.2 ± 0.08 ^bA^	2.19 ± 0.08 ^aA^	4.25 ± 0.13 ^cA^	3.36 ± 0.08 ^bA^	2.27 ± 0.16 ^aA^	4.29 ± 0.33 ^cA^	3.40 ± 0.23 ^bA^	2.32 ± 0.17 ^aA^
Quercetin	1.69 ± 0.10 ^aA^	1.61 ± 0.04 ^aA^	1.57 ± 0.06 ^aA^	1.59 ± 0.05 ^aA^	1.55 ± 0.08 ^aA^	1.50 ± 0.06 ^aA^	1.62 ± 0.06 ^aA^	1.58 ± 0.06 ^aA^	1.54 ± 0.08 ^aA^	1.63 ± 0.10 ^aA^	1.60 ± 0.08 ^aA^	1.58 ± 0.01 ^aA^
Luteolin	2.65 ± 0.03 ^bA^	2.37 ± 0.06 ^aA^	2.25 ± 0.08 ^aA^	2.49 ± 0.05 ^aA^	2.43 ± 0.09 ^aA^	2.35 ± 0.15 ^aA^	2.51 ± 0.07 ^aA^	2.46 ± 0.11 ^aA^	2.42 ± 0.11 ^aA^	2.53 ± 0.11 ^aA^	2.49 ± 0.08 ^aA^	2.47 ± 0.09 ^aA^
Salicylic Acid	0.69 ± 0.03 ^bA^	0.57 ± 0.03 ^aA^	0.51 ± 0.03 ^aA^	0.67 ± 0.04 ^bA^	0.6 ± 0.01 ^abAB^	0.55 ± 0.04 ^aAB^	0.68 ± 0.03 ^bA^	0.64 ± 0.03 ^abB^	0.57 ± 0.01 ^aAB^	0.69 ± 0.05 ^aA^	0.66 ± 0.01 ^aB^	0.60 ± 0.03 ^aC^

Lowercase letters (a,b,c) indicate statistical differences between storage times (0, 45, 90) within the same sample group. Uppercase letters (A,B,C) indicate statistical differences observed between different groups at the same storage time.

**Table 9:** Phenolic compounds of KY olive oil and microcapsules during storage (0, 45, 90 days) (mg/kg).

Phenolic Compounds (µg/kg)	KY			KY (1:1)			KY (1:4)			KY (1:10)		
	0. Day	45. Day	90. Day	0. Day	45. Day	90. Day	0. Day	45. Day	90. Day	0. Day	45. Day	90. Day
Vanilic Acid	14.02 ± 0.37 ^bA^	13.76 ± 0.28 ^bA^	8.67 ± 0.21 ^aA^	13.64 ± 0.42 ^bA^	13.05 ± 0.28 ^bA^	11.78 ± 0.28 ^aB^	13.74 ± 0.40 ^bA^	13.46 ± 0.42 ^abA^	12.1 ± 0.62 ^aB^	13.82 ± 0.59 ^aA^	13.52 ± 0.45 ^aA^	12.55 ± 0.30 ^aB^
Acetohydroxamic Acid	2.72 ± 0.14 ^bA^	2.60 ± 0.07 ^abA^	2.28 ± 0.08 ^aA^	2.68 ± 0.14 ^aA^	2.52 ± 0.14 ^aA^	2.31 ± 0.07 ^aA^	2.60 ± 0.08 ^bA^	2.42 ± 0.10 ^aA^	2.38 ± 0.14 ^aA^	2.63 ± 0.10 ^aA^	2.57 ± 0.17 ^aA^	2.45 ± 0.11 ^aA^
Kaempferol	2.24 ± 0.08 ^cA^	2.01 ± 0.07 ^bA^	1.69 ± 0.05 ^aA^	2.20 ± 0.14 ^bA^	1.83 ± 0.11 ^aA^	1.74 ± 0.03 ^aA^	2.16 ± 0.17 ^aA^	1.99 ± 0.19 ^aA^	1.80 ± 0.14 ^aA^	2.18 ± 0.11 ^bA^	2.01 ± 0.04 ^abA^	1.87 ± 0.11 ^aA^
Chlorogenic Acid	3.85 ± 0.16 ^cA^	3.3 ± 0.06 ^bA^	2.55 ± 0.16 ^aA^	3.52 ± 0.17 ^bA^	3.25 ± 0.24 ^abA^	2.76 ± 0.11 ^aAB^	3.58 ± 0.20 ^cA^	3.34 ± 0.13 ^abA^	2.90 ± 0.19 ^aAB^	3.58 ± 0.20 ^aA^	3.43 ± 0.08 ^aA^	3.08 ± 0.18 ^aB^
Fumaric Acid	1.64 ± 0.08 ^bA^	1.50 ± 0.07 ^bA^	1.02 ± 0.08 ^aA^	1.59 ± 0.07 ^bA^	1.53 ± 0.11 ^bA^	1.17 ± 0.07 ^aAB^	1.57 ± 0.05 ^bA^	1.53 ± 0.07 ^bA^	1.26 ± 0.01 ^aB^	1.60 ± 0.10 ^aA^	1.57 ± 0.08 ^aA^	1.34 ± 0.06 ^aB^
Caffeic Acid	0.59 ± 0.01 ^cA^	0.17 ± 0.01 ^bA^	0.11 ± 0.01 ^aA^	0.54 ± 0.00 ^cA^	0.45 ± 0.02 ^bA^	0.27 ± 0.02 ^aB^	0.53 ± 0.04 ^bA^	0.46 ± 0.02 ^aA^	0.33 ± 0.01 ^aC^	0.58 ± 0.02 ^cA^	0.48 ± 0.01 ^bA^	0.41 ± 0.03 ^aD^
Hydroxycinamic Acid	4.47 ± 0.12 ^cA^	3.14 ± 0.17 ^bA^	2.08 ± 0.14 ^aA^	4.34 ± 0.16 ^cA^	3.32 ± 0.11 ^bA^	2.25 ± 0.10 ^aA^	4.35 ± 0.30 ^cA^	3.46 ± 0.20 ^bA^	2.35 ± 0.16 ^aA^	4.39 ± 0.11 ^cA^	3.55 ± 0.11 ^bA^	2.43 ± 0.16 ^aA^
Quercetin	1.63 ± 0.10 ^aA^	1.58 ± 0.04 ^aA^	1.56 ± 0.03 ^aA^	1.61 ± 0.06 ^aA^	1.57 ± 0.04 ^aA^	1.55 ± 0.10 ^aA^	1.59 ± 0.05 ^aA^	1.58 ± 0.08 ^aA^	1.54 ± 0.08 ^aA^	1.60 ± 0.13 ^aA^	1.57 ± 0.09 ^aA^	1.56 ± 0.05 ^aA^
Luteolin	2.60 ± 0.18 ^aA^	2.43 ± 0.09 ^aA^	2.34 ± 0.12 ^aA^	2.53 ± 0.16 ^aA^	2.48 ± 0.08 ^aA^	2.41 ± 0.06 ^aA^	2.57 ± 0.16 ^aA^	2.54 ± 0.14 ^aA^	2.47 ± 0.13 ^aA^	2.58 ± 0.17 ^aA^	2.55 ± 0.11 ^aA^	2.50 ± 0.11 ^aA^
Salicylic Acid	0.67 ± 0.03 ^bA^	0.55 ± 0.02 ^aA^	0.47 ± 0.03 ^aA^	0.65 ± 0.04 ^cA^	0.58 ± 0.04 ^abA^	0.51 ± 0.02 ^aAB^	0.66 ± 0.04 ^aA^	0.63 ± 0.03 ^aA^	0.58 ± 0.04 ^aAB^	0.66 ± 0.01 ^aA^	0.63 ± 0.03 ^aA^	0.59 ± 0.06 ^aB^

Lowercase letters (a,b,c) indicate statistical differences between storage times (0, 45, 90) within the same sample group. Uppercase letters (A,B,C,D) indicate statistical differences observed between different groups at the same storage time.

**Table 10:** Antioxidant activity (mM TE/kg) of olive oil and microcapsules during storage (0, 45, 90 days).

Antioxidant Activity (mM TE/kg)					
Sample	Storage Time (Day)	Mean ± Std Dev	Sample	Storage Time (Day)	Mean ± Std Dev
NY	0	0.77 ± 0.01 ^cAB^	KY	0	0.79 ± 0.01 ^cA^
	45	0.74 ± 0.01 ^bA^		45	0.73 ± 0.01 ^bA^
	90	0.68 ± 0.01 ^aA^		90	0.68 ± 0.01 ^aA^
NY (1:1)	0	0.76 ± 0.01 ^cAB^	KY (1:1)	0	0.78 ± 0.02 ^cA^
	45	0.73 ± 0.01 ^bA^		45	0.74 ± 0.01 ^bA^
	90	0.70 ± 0.01 ^aA^		90	0.71 ± 0.01 ^aB^
NY (1:4)	0	0.76 ± 0.00 ^bA^	KY (1:4)	0	0.76 ± 0.01 ^bB^
	45	0.74 ± 0.01 ^aA^		45	0.73 ± 0.01 ^aA^
	90	0.72 ± 0.01 ^aAB^		90	0.71 ± 0.01 ^aB^
NY (1:10)	0	0.78 ± 0.01 ^bB^	KY (1:10)	0	0.75 ± 0.01 ^bB^
	45	0.75 ± 0.01 ^aA^		45	0.74 ± 0.01 ^bA^
	90	0.74 ± 0.01 ^aB^		90	0.72 ± 0.00 ^aB^

Lowercase letters (a,b,c) in the same column indicate statistical differences between storage times (0, 45, 90) within the same sample group. Uppercase letters (A,B) in the same column indicate statistical differences observed between different groups at the same storage time.

## Data Availability

The original contributions presented in the study are included in the article, further inquiries can be directed to the corresponding authors.
